# Crystal structure of 1-amino-2-oxo-2,5,6,7,8,9-hexa­hydro-1*H*-cyclo­hepta­[*b*]pyridine-3-carbo­nitrile

**DOI:** 10.1107/S2056989016012196

**Published:** 2016-08-02

**Authors:** Galal H. Elgemeie, Peter G. Jones

**Affiliations:** a Chemistry Department, Faculty of Science, Helwan University, Cairo, Egypt; b Institut für Anorganische und Analytische Chemie, Technische Universität Braunschweig, Postfach 3329, D-38023 Braunschweig, Germany

**Keywords:** crystal structure, cyclo­hepta­[*b*]pyridine, carbo­nitrile, N—NH_2_ group, tautomer, N—H⋯O hydrogen bonding

## Abstract

In the title compound, C_11_H_13_N_3_O, the seven-membered ring adopts a conformation such that the three atoms not involved in the aromatic plane lie on the same side of that plane. One hydrazinic H atom forms an intra­molecular hydrogen bond to the O atom; the other forms a classical inter­molecular hydrogen bond N—H⋯O, which combines with a ‘weak’ H_ar_⋯O inter­action to build up double layers of mol­ecules parallel to the *bc* plane.

## Abstract 2

In the title compound the seven-membered ring adopts a conformation such that the three atoms not involved in the aromatic plane lie on the same side of that plane. One hydrazinic H atom forms an intra­molecular hydrogen bond to the O atom; the other forms a classical inter­molecular hydrogen bond N—H⋯O, which combines with a ‘weak’ H_ar_⋯O inter­action to build up double layers of mol­ecules parallel to the *bc* plane.

## Chemical context   

We have recently described various novel approaches for the synthesis of a new class of N-substituted amino derivatives of pyridines and pyrimidines (Elgemeie, Salah *et al.*, 2015 ▸; Elgemeie *et al.*, 2016 ▸). These compounds are important as pyrimidine ring systems that are not nucleoside analogs, and are inter­esting as anti­metabolic agents (Elgemeie & Hamed, 2014 ▸; Elgemeie & Abd Elaziz, 2015 ▸). They have a greater selectivity for a broader range of human tumors, hence our inter­est in this class of compounds (Elgemeie, Abou-Zeid *et al.*, 2015 ▸; Elgemeie, Mohamed *et al.*, 2015 ▸). 
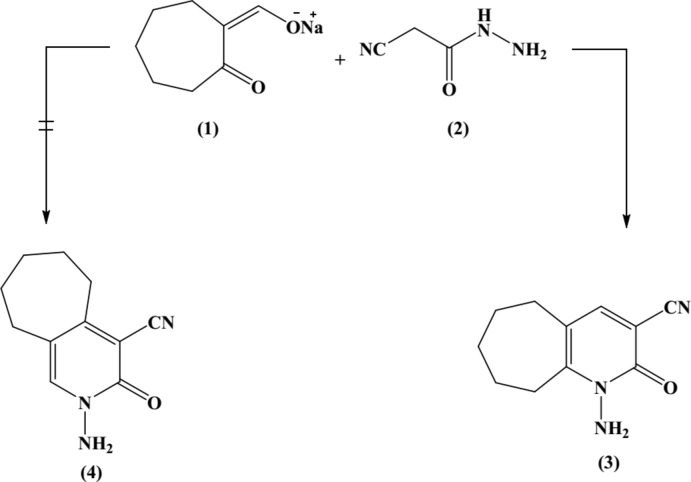


We report here a novel one-step synthesis of a cyclo­heptane-ring-fused *N*-amino-2-pyridone derivative by reaction of the sodium salt of 2-(hy­droxy­methyl­ene)-1-cyclo­hepta­none (1) with a cyano­acetohydrazide (2). Thus, (1) reacted with (2) in piperidine acetate to give a product of mol­ecular formula C_11_H_13_N_3_O (*M* ^+^ = 203), for which two isomeric structures, (3) and (4), seemed possible, corres­ponding to two possible modes of cyclization. Spectroscopic data cannot differentiate between these structures, and therefore the crystal structure was determined, confirming the exclusive presence of tautomer (3) in the solid state. The formation of (3) from the reaction of (1) and (2) is assumed to proceed *via* initial addition of the active methyl­ene carbon atom of (2) to the formyl group of (1) to give the favoured, kinetically controlled product (3). The ^1^H NMR spectra of the product revealed the presence of an N—NH_2_ group at δ = 6.4 p.p.m. and a pyridine H-4 at 7.8 p.p.m. in solution.

## Structural commentary   

**Figure 1:**
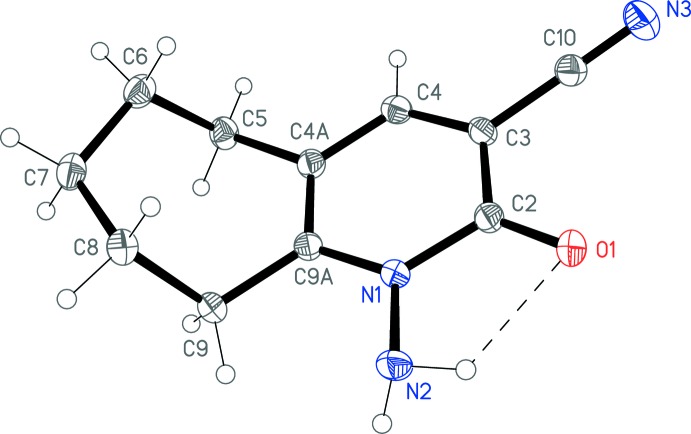
The mol­ecular structure of the title compound, with atom labelling. Displacement ellipsoids are drawn at the 50% probability level. The intra­molecular N—H⋯O hydrogen bond is shown as a dashed line (see Table 1 ▸)

The structure of the title compound is shown in Fig. 1 ▸ and confirms the presence of tautomer (3) in the solid state. Mol­ecular dimensions [*e.g.* the hydrazinic N1—N2 bond length of 1.4201 (15) Å] may be regarded as normal; an extensive structural investigation of alkyl-substituted 3-cyano-2-pyridones (with an unsubstituted NH function in the ring) was published by Fischer *et al.* (2004 ▸). The seven-membered ring adopts a conformation such that all three atoms C6, C7 and C8 lie to the same side of the plane formed by the pyridone ring together with C5 and C9; the respective deviations from this plane are 1.480 (2), 1.616 (3) and 1.470 (2) Å.

## Supra­molecular features   

**Figure 2:**
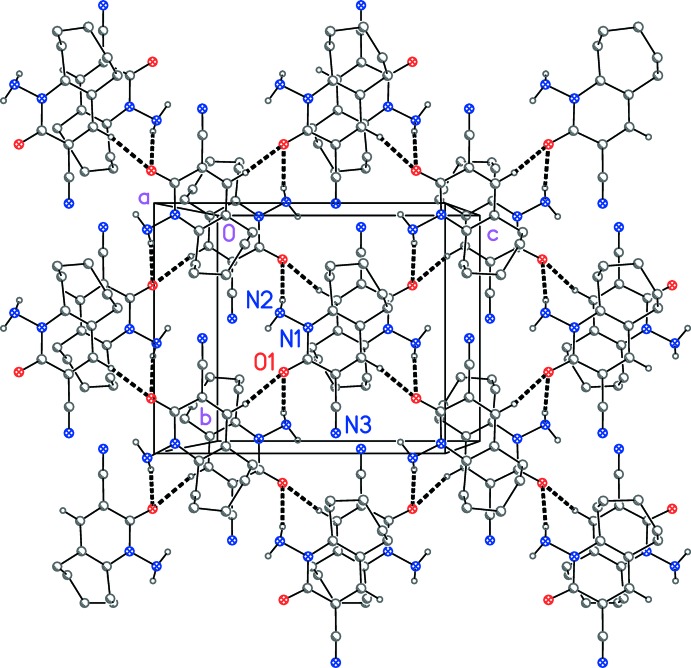
Crystal packing of the title compound, viewed approximately normal to the *bc* plane. Dashed lines indicate the hydrogen bonds (see Table 1 ▸), and for clarity H atoms not involved in hydrogen bonding have been omitted.

The classical hydrogen-bond donor N1—H01 is only involved in intra­molecular hydrogen bonding (Fig. 1 ▸ and Table 1 ▸). The second such donor N1—H02 forms a classical hydrogen bond to the acceptor O1 of a neighbouring mol­ecule related by the 2_1_ screw axis. Additionally, the ‘weak’ but quite short hydrogen bond C4—H4⋯O1 links mol­ecules related by the *c* glide plane. The overall effect is to build up double layers of mol­ecules (Fig. 2 ▸ and Table 1 ▸) parallel to the *bc* plane, in which the top and bottom mol­ecules of the layer are related by inversion.

## Database survey   

A search of the Cambridge Structural Database (CSD, Version 5.37, last update May 2016; Groom *et al.*, 2016 ▸) revealed four other examples of the cyclo­hepta­[*b*]pyridin-2-one ring system: refcodes AHEQAF (Elgemeie *et al.*, 2002 ▸), ATUYAP and IBATUB (Albov *et al.*, 2004*a* ▸,**b* ▸)* and QAHLOB (Fischer *et al.*, 2004 ▸).

## Synthesis and crystallization   

A solution of the sodium salt of 2-(hy­droxy­methyl­ene)-1-cyclo­hepta­none [(1); 1.60 g, 0.01 mol], *N*-cyano­acetohydrazide [(2); 0.09 g, 0.01 mol] and piperidine acetate (1 ml) in water (30 ml) and ethanol (30 ml) was refluxed for 10 min. Acetic acid (1.5 ml) was added to the hot solution. The precipitated solid was collected by filtration and crystallized from ethanol, giving colourless plate-like crystals (yield 85%, m.p. 508 K).

## Refinement   

Crystal data, data collection and structure refinement details are summarized in Table 2 ▸. The NH hydrogens were located in a difference Fourier map and freely refined. The C-bound H atoms were included using a riding model starting from calculated positions: C—H = 0.95–0.99 Å with *U*_iso_(H) = 1.2*U*_eq_(C).

## Supplementary Material

Crystal structure: contains datablock(s) I, global. DOI: 10.1107/S2056989016012196/su5315sup1.cif

Structure factors: contains datablock(s) I. DOI: 10.1107/S2056989016012196/su5315Isup2.hkl

Supporting information file. DOI: 10.1107/S2056989016012196/su5315Isup3.cml

CCDC reference: 1496294

Additional supporting information: crystallographic information; 3D view; checkCIF report

## Figures and Tables

**Table 1:** Hydrogen-bond geometry (Å, °)

*D*—H⋯*A*	*D*—H	H⋯*A*	*D*⋯*A*	*D*—H⋯*A*
N2—H01⋯O1	0.90 (2)	2.05 (2)	2.6255 (15)	120.4 (16)
N2—H02⋯O1^i^	0.91 (2)	2.16 (2)	3.0225 (15)	158.2 (17)
C4—H4⋯O1^ii^	0.95	2.45	3.2105 (16)	137
C9—H9*A*⋯O1^i^	0.99	2.63	3.4903 (16)	146

Symmetry codes: (i) 
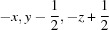
; (ii) 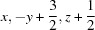.

**Table 2:** Experimental details

Crystal data	
Chemical formula	C_11_H_13_N_3_O
*M*_r_	203.24
Crystal system, space group	Monoclinic, *P*2_1_/*c*
Temperature (K)	100
*a*, *b*, *c* (Å)	8.5680 (4), 10.0475 (4), 11.6778 (5)
β (°)	103.272 (4)
*V* (Å^3^)	978.46 (7)
*Z*	4
Radiation type	Cu *K*α
μ (mm^−1^)	0.74
Crystal size (mm)	0.10 × 0.10 × 0.05

Data collection	
Diffractometer	Oxford Diffraction Xcalibur Atlas Nova
Absorption correction	Multi-scan (*CrysAlis PRO*; Agilent, 2010 ▸)
*T*_min_, *T*_max_	0.795, 1.000
No. of measured, independent and observed [*I* > 2σ(*I*)] reflections	19833, 2046, 1674
*R*_int_	0.062
(sin θ/λ)_max_ (Å^−1^)	0.630

Refinement	
*R*[*F* ^2^ > 2σ(*F* ^2^)], *wR*(*F* ^2^), *S*	0.039, 0.109, 1.06
No. of reflections	2046
No. of parameters	144
H-atom treatment	H atoms treated by a mixture of independent and constrained refinement
Δρ_max_, Δρ_min_ (e Å^−3^)	0.28, −0.30

Computer programs: (*CrysAlis PRO*; Agilent, 2010 ▸), *SHELXS97* and *SHELXL97* (Sheldrick, 2008 ▸) and *XP* (Siemens, 1994 ▸).

## supplementary crystallographic information

### Crystal data

**Table:**

C_11_H_13_N_3_O	*F*(000) = 432
*M**_r_* = 203.24	*D*_x_ = 1.380 Mg m^−^^3^
Monoclinic, *P*2_1_/*c*	Cu *K*α radiation, λ = 1.54184 Å
Hall symbol: -P 2ybc	Cell parameters from 6583 reflections
*a* = 8.5680 (4) Å	θ = 3.9–76.1°
*b* = 10.0475 (4) Å	µ = 0.74 mm^−^^1^
*c* = 11.6778 (5) Å	*T* = 100 K
β = 103.272 (4)°	Plate, colourless
*V* = 978.46 (7) Å^3^	0.10 × 0.10 × 0.05 mm
*Z* = 4	

### Data collection

**Table:**

Oxford Diffraction Xcalibur Atlas Nova diffractometer	2046 independent reflections
Radiation source: sealed X-ray tube, Nova (Cu) X-ray Source	1674 reflections with *I* > 2σ(*I*)
Mirror monochromator	*R*_int_ = 0.062
Detector resolution: 10.3543 pixels mm^-1^	θ_max_ = 76.1°, θ_min_ = 5.3°
ω–scan	*h* = −10→10
Absorption correction: multi-scan (CrysAlis PRO; Agilent, 2010)	*k* = −12→12
*T*_min_ = 0.795, *T*_max_ = 1.000	*l* = −14→14
19833 measured reflections	

### Refinement

**Table:**

Refinement on *F*^2^	Primary atom site location: structure-invariant direct methods
Least-squares matrix: full	Secondary atom site location: difference Fourier map
*R*[*F*^2^ > 2σ(*F*^2^)] = 0.039	Hydrogen site location: inferred from neighbouring sites
*wR*(*F*^2^) = 0.109	H atoms treated by a mixture of independent and constrained refinement
*S* = 1.06	*w* = 1/[σ^2^(*F*_o_^2^) + (0.0602*P*)^2^ + 0.2053*P*] where *P* = (*F*_o_^2^ + 2*F*_c_^2^)/3
2046 reflections	(Δ/σ)_max_ < 0.001
144 parameters	Δρ_max_ = 0.28 e Å^−^^3^
0 restraints	Δρ_min_ = −0.30 e Å^−^^3^

### Special details

**Table:**

Geometry. All esds (except the esd in the dihedral angle between two l.s. planes) are estimated using the full covariance matrix. The cell esds are taken into account individually in the estimation of esds in distances, angles and torsion angles; correlations between esds in cell parameters are only used when they are defined by crystal symmetry. An approximate (isotropic) treatment of cell esds is used for estimating esds involving l.s. planes. Least-squares planes (x,y,z in crystal coordinates) and deviations from them (* indicates atom used to define plane) 8.5275 (0.0007) x + 0.7466 (0.0052) y - 3.3783 (0.0058) z = 0.3993 (0.0040) * -0.0117 (0.0008) N1 * 0.0031 (0.0009) C2 * 0.0072 (0.0009) C3 * -0.0090 (0.0009) C4 * 0.0007 (0.0009) C4A * 0.0097 (0.0009) C9A 1.4803 (0.0024) C6 1.6155 (0.0026) C7 1.4700 (0.0023) C8 Rms deviation of fitted atoms = 0.0079
Refinement. Refinement of F^2^ against ALL reflections. The weighted R-factor wR and goodness of fit S are based on F^2^, conventional R-factors R are based on F, with F set to zero for negative F^2^. The threshold expression of F^2^ > 2sigma(F^2^) is used only for calculating R-factors(gt) etc. and is not relevant to the choice of reflections for refinement. R-factors based on F^2^ are statistically about twice as large as those based on F, and R- factors based on ALL data will be even larger.

### Fractional atomic coordinates and isotropic or equivalent isotropic displacement parameters (Å^2^)

**Table:**

	*x*	*y*	*z*	*U*_iso_*/*U*_eq_	
N1	0.14967 (13)	0.49677 (10)	0.37289 (9)	0.0161 (3)	
C2	0.13750 (15)	0.63589 (13)	0.36852 (11)	0.0168 (3)	
C3	0.17684 (15)	0.69947 (13)	0.48064 (12)	0.0168 (3)	
C4	0.22210 (15)	0.62730 (13)	0.58375 (11)	0.0173 (3)	
H4	0.2451	0.6727	0.6571	0.021*	
C4A	0.23457 (16)	0.48899 (13)	0.58178 (11)	0.0169 (3)	
C5	0.29860 (17)	0.41294 (13)	0.69430 (11)	0.0197 (3)	
H5A	0.2243	0.3391	0.7000	0.024*	
H5B	0.3025	0.4729	0.7622	0.024*	
C6	0.46697 (17)	0.35590 (14)	0.70100 (12)	0.0218 (3)	
H6A	0.5315	0.4231	0.6703	0.026*	
H6B	0.5197	0.3395	0.7845	0.026*	
C7	0.46682 (17)	0.22636 (14)	0.63197 (12)	0.0212 (3)	
H7A	0.5787	0.1942	0.6447	0.025*	
H7B	0.4055	0.1587	0.6651	0.025*	
C8	0.39649 (16)	0.23537 (13)	0.49953 (12)	0.0199 (3)	
H8A	0.4085	0.1478	0.4637	0.024*	
H8B	0.4596	0.3008	0.4656	0.024*	
C9	0.21817 (16)	0.27618 (13)	0.46552 (12)	0.0186 (3)	
H9A	0.1708	0.2467	0.3841	0.022*	
H9B	0.1597	0.2312	0.5185	0.022*	
C9A	0.19855 (15)	0.42414 (13)	0.47385 (11)	0.0159 (3)	
C10	0.17377 (16)	0.84204 (14)	0.48160 (12)	0.0200 (3)	
N2	0.12201 (15)	0.43233 (12)	0.26182 (10)	0.0207 (3)	
H01	0.090 (2)	0.500 (2)	0.2110 (18)	0.036 (5)*	
H02	0.037 (2)	0.376 (2)	0.2551 (16)	0.030 (5)*	
N3	0.17469 (17)	0.95620 (12)	0.48127 (12)	0.0298 (3)	
O1	0.09637 (12)	0.69230 (9)	0.27097 (8)	0.0208 (2)	

### Atomic displacement parameters (Å^2^)

**Table:**

	*U*^11^	*U*^22^	*U*^33^	*U*^12^	*U*^13^	*U*^23^
N1	0.0174 (6)	0.0137 (5)	0.0162 (5)	−0.0004 (4)	0.0016 (4)	−0.0012 (4)
C2	0.0134 (6)	0.0151 (6)	0.0214 (7)	0.0001 (4)	0.0028 (5)	0.0014 (5)
C3	0.0150 (6)	0.0129 (6)	0.0223 (6)	−0.0009 (4)	0.0037 (5)	−0.0012 (5)
C4	0.0154 (6)	0.0171 (6)	0.0199 (6)	−0.0005 (5)	0.0047 (5)	−0.0024 (5)
C4A	0.0153 (6)	0.0162 (6)	0.0192 (6)	0.0000 (5)	0.0036 (5)	0.0012 (5)
C5	0.0240 (7)	0.0182 (6)	0.0170 (6)	0.0015 (5)	0.0051 (5)	0.0018 (5)
C6	0.0212 (7)	0.0241 (7)	0.0182 (6)	0.0018 (5)	0.0006 (5)	0.0020 (5)
C7	0.0197 (7)	0.0204 (7)	0.0229 (7)	0.0036 (5)	0.0037 (5)	0.0044 (5)
C8	0.0199 (7)	0.0182 (6)	0.0215 (6)	0.0026 (5)	0.0048 (5)	0.0005 (5)
C9	0.0193 (7)	0.0137 (6)	0.0218 (6)	−0.0002 (5)	0.0026 (5)	−0.0003 (5)
C9A	0.0133 (6)	0.0145 (6)	0.0195 (6)	−0.0006 (4)	0.0030 (5)	0.0008 (4)
C10	0.0183 (6)	0.0182 (7)	0.0227 (6)	0.0002 (5)	0.0033 (5)	−0.0007 (5)
N2	0.0272 (7)	0.0176 (6)	0.0154 (5)	−0.0018 (5)	0.0006 (5)	−0.0033 (4)
N3	0.0340 (8)	0.0182 (6)	0.0366 (7)	0.0000 (5)	0.0068 (6)	−0.0004 (5)
O1	0.0237 (5)	0.0170 (4)	0.0199 (5)	0.0012 (4)	0.0012 (4)	0.0039 (4)

### Geometric parameters (Å, º)

**Table:**

N1—C9A	1.3680 (16)	C10—N3	1.1471 (19)
N1—C2	1.4017 (16)	C4—H4	0.9500
N1—N2	1.4201 (15)	C5—H5A	0.9900
C2—O1	1.2486 (16)	C5—H5B	0.9900
C2—C3	1.4260 (18)	C6—H6A	0.9900
C3—C4	1.3826 (18)	C6—H6B	0.9900
C3—C10	1.4328 (18)	C7—H7A	0.9900
C4—C4A	1.3943 (18)	C7—H7B	0.9900
C4A—C9A	1.3892 (18)	C8—H8A	0.9900
C4A—C5	1.5101 (17)	C8—H8B	0.9900
C5—C6	1.5375 (19)	C9—H9A	0.9900
C6—C7	1.5308 (19)	C9—H9B	0.9900
C7—C8	1.5283 (18)	N2—H01	0.90 (2)
C8—C9	1.5432 (18)	N2—H02	0.91 (2)
C9—C9A	1.5018 (17)		
			
C9A—N1—C2	124.66 (11)	C4A—C5—H5B	109.1
C9A—N1—N2	119.86 (10)	C6—C5—H5B	109.1
C2—N1—N2	115.22 (10)	H5A—C5—H5B	107.8
O1—C2—N1	119.27 (11)	C7—C6—H6A	108.8
O1—C2—C3	126.31 (12)	C5—C6—H6A	108.8
N1—C2—C3	114.42 (11)	C7—C6—H6B	108.8
C4—C3—C2	121.64 (12)	C5—C6—H6B	108.8
C4—C3—C10	121.30 (12)	H6A—C6—H6B	107.7
C2—C3—C10	117.01 (12)	C8—C7—H7A	108.3
C3—C4—C4A	121.04 (12)	C6—C7—H7A	108.3
C9A—C4A—C4	118.72 (12)	C8—C7—H7B	108.3
C9A—C4A—C5	120.85 (12)	C6—C7—H7B	108.3
C4—C4A—C5	120.26 (12)	H7A—C7—H7B	107.4
C4A—C5—C6	112.46 (11)	C7—C8—H8A	108.7
C7—C6—C5	113.75 (11)	C9—C8—H8A	108.7
C8—C7—C6	115.79 (11)	C7—C8—H8B	108.7
C7—C8—C9	114.41 (11)	C9—C8—H8B	108.7
C9A—C9—C8	111.42 (11)	H8A—C8—H8B	107.6
N1—C9A—C4A	119.48 (12)	C9A—C9—H9A	109.3
N1—C9A—C9	119.26 (11)	C8—C9—H9A	109.3
C4A—C9A—C9	121.22 (12)	C9A—C9—H9B	109.3
N3—C10—C3	178.28 (16)	C8—C9—H9B	109.3
C3—C4—H4	119.5	H9A—C9—H9B	108.0
C4A—C4—H4	119.5	N1—N2—H01	102.6 (13)
C4A—C5—H5A	109.1	N1—N2—H02	108.8 (12)
C6—C5—H5A	109.1	H01—N2—H02	107.1 (17)
			
C9A—N1—C2—O1	177.72 (12)	C4A—C5—C6—C7	−80.60 (15)
N2—N1—C2—O1	3.59 (17)	C5—C6—C7—C8	61.27 (16)
C9A—N1—C2—C3	−1.60 (18)	C6—C7—C8—C9	−61.68 (16)
N2—N1—C2—C3	−175.73 (11)	C7—C8—C9—C9A	80.91 (14)
O1—C2—C3—C4	−179.57 (13)	C2—N1—C9A—C4A	2.30 (19)
N1—C2—C3—C4	−0.30 (18)	N2—N1—C9A—C4A	176.17 (12)
O1—C2—C3—C10	−2.1 (2)	C2—N1—C9A—C9	−175.43 (12)
N1—C2—C3—C10	177.22 (11)	N2—N1—C9A—C9	−1.55 (18)
C2—C3—C4—C4A	1.5 (2)	C4—C4A—C9A—N1	−0.99 (18)
C10—C3—C4—C4A	−175.93 (13)	C5—C4A—C9A—N1	−176.19 (12)
C3—C4—C4A—C9A	−0.82 (19)	C4—C4A—C9A—C9	176.68 (12)
C3—C4—C4A—C5	174.41 (12)	C5—C4A—C9A—C9	1.48 (19)
C9A—C4A—C5—C6	66.67 (16)	C8—C9—C9A—N1	109.73 (13)
C4—C4A—C5—C6	−108.45 (14)	C8—C9—C9A—C4A	−67.96 (16)

### Hydrogen-bond geometry (Å, º)

**Table:**

*D*—H···*A*	*D*—H	H···*A*	*D*···*A*	*D*—H···*A*
N2—H01···O1	0.90 (2)	2.05 (2)	2.6255 (15)	120.4 (16)
N2—H02···O1^i^	0.91 (2)	2.16 (2)	3.0225 (15)	158.2 (17)
C4—H4···O1^ii^	0.95	2.45	3.2105 (16)	137
C9—H9*A*···O1^i^	0.99	2.63	3.4903 (16)	146

Symmetry codes: (i) −*x*, *y*−1/2, −*z*+1/2; (ii) *x*, −*y*+3/2, *z*+1/2.
